# Predicting pathogenicity for novel hearing loss mutations based on genetic and protein structure approaches

**DOI:** 10.1038/s41598-021-04081-2

**Published:** 2022-01-07

**Authors:** Paula I. Buonfiglio, Carlos D. Bruque, Vanesa Lotersztein, Leonela Luce, Florencia Giliberto, Sebastián Menazzi, Liliana Francipane, Bibiana Paoli, Ernesto Goldschmidt, Ana Belén Elgoyhen, Viviana Dalamón

**Affiliations:** 1 Laboratory of Physiology and Genetics of Hearing. Instituto de Investigaciones en Ingeniería Genética y Biología Molecular “Dr. Héctor Torres”, Consejo Nacional de Investigaciones Científicas y Técnicas, INGEBI/CONICET, Vuelta de Obligado 2490- (C1428ADN), Ciudad Autónoma de Buenos Aires, Argentina; 2 Unidad de Conocimiento Traslacional Hospitalaria Patagónica, Hospital de Alta Complejidad SAMIC - El Calafate, Provincia de Santa Cruz, Argentina; 3 Servicio de Genética, Hospital Militar Central “Dr. Cosme Argerich”, C1426 Ciudad Autónoma de Buenos Aires, Argentina; 4 Laboratorio de Distrofinopatías. Cátedra de Genética, Facultad de Farmacia y Bioquímica, Universidad de Buenos Aires, C1113AAD Ciudad Autónoma de Buenos Aires, Argentina; 5 División Genética, Hospital de Clínicas “José de San Martín”, C1120AAR Ciudad Autónoma de Buenos Aires, Argentina; 6 Sector de Otorrinolaringología Infantil, Hospital de Clínicas “José de San Martín”, C1120AAR Ciudad Autónoma de Buenos Aires, Argentina; 7 Laboratorio Diagnogen, Ciudad Autónoma de Buenos Aires, Argentina; 8 Instituto de Farmacología, Facultad de Medicina, Universidad de Buenos Aires, C1121ABG Ciudad Autónoma de Buenos Aires, Argentina

**Keywords:** Computational biology and bioinformatics, Genetics, Molecular biology, Diseases, Medical research, Molecular medicine

## Abstract

Hearing loss is a heterogeneous disorder. Identification of causative mutations is demanding due to genetic heterogeneity. In this study, we investigated the genetic cause of sensorineural hearing loss in patients with severe/profound deafness. After the exclusion of *GJB2-GJB6* mutations, we performed whole exome sequencing in 32 unrelated Argentinean families. Mutations were detected in 16 known deafness genes in 20 patients: *ACTG*1, *ADGRV*1 (*GPR*98), *CDH*23, *COL4*A3, *COL*4A5, *DFNA*5 (*GSDDE*), *EYA*4, *LARS*2, *LOXHD*1, *MITF*, *MYO*6, *MYO*7A, *TECTA, TMPRSS*3, *USH*2*A* and *WSF*1*.* Notably, 11 variants affecting 9 different non-*GJB2* genes resulted novel: c.12829C > T, p.(Arg4277*) in ADGRV1; c.337del, p.(Asp109*) and c.3352del, p.(Gly1118Alafs*7) in CDH23; c.3500G > *A, p.(Gly1167Glu) in COL4A3; c.1183C* > *T, p.(Pro395Ser) and c.1759C* > *T, p.(Pro587Ser) in COL4A5; c.580* + *2 T* > C in EYA4; c.1481dup, p.(Leu495Profs*31) in LARS2; c.1939 T > *C, p.(Phe647Leu), in MYO6; c.733C* > T, p.(Gln245*) in MYO7A and c.242C > G, p.(Ser81*) in TMPRSS3 genes. To predict the effect of these variants, novel protein modeling and protein stability analysis were employed. These results highlight the value of whole exome sequencing to identify candidate variants, as well as bioinformatic strategies to infer their pathogenicity.

## Introduction

Deafness affects approximately 1 out of 500–1000 newborns and is mainly of genetic origin. The genetic causes that lead to hearing loss can be categorized into syndromic and nonsyndromic conditions, which constitute 30% and 70% of the genetic forms, respectively^1^. Over 400 distinct syndromes that include hearing impairment are listed in Online Mendelian Inheritance in Man (OMIM), the most common including Alport, Pendred, Usher and Waardenburg^1–3^. Despite the wide genetic heterogeneity of Nonsyndromic autosomal recessive hearing loss, a few mutations in the *GJB*2 and *GJB*6 genes (encoding connexin-26 and 30, respectively) account for nearly 50% of the cases in the Mediterranean population^4–8^. To date, approximately 100 genes and more than 1000 mutations causing nonsyndromic deafness have been reported: more than 72 genes associated with autosomal recessive nonsyndromic deafness (named locus DFNB1 to 108) and nearly 44 with autosomal dominant forms, named locus DFNA1 to 73 (http://hereditaryhearingloss.org/). Molecular diagnosis of hearing loss (HL) remains a challenge due to the high number of genes involved. Genetic screening consists of analyzing many and sometimes long genes for mutations, making conventional methods (e.g., Sanger sequencing) expensive and time-consuming^9^*.* Except for mutations in *GJB*2, most deafness mutations are seen in only a single or few families^10^. Moreover, in a limited number of cases, the characteristics of hearing impairment (audiogram shape, hearing impairment onset and progression or family history) pinpoint the gene to test. Therefore, an approach that assays for most or all of the known genes at the same time would ease and accelerate diagnosis. Whole-exome sequencing (WES) has thus become an efficient and cost-effective alternative approach for molecular diagnosis of hearing impairment. After the exclusion of *GJB*2 and *GJB*6 in patients, new genes and new genetic variants in reported genes have been described using WES in the research and clinical molecular diagnosis of syndromic and nonsyndromic forms of hearing impairment. Moreover, WES is gradually being integrated into the routine genetic diagnostics of Mendelian diseases, including hereditary HL^11–14^. However, the follow-up of novel variants, in particular missense changes, which can lead to a spectrum of phenotypes and unequivocal genotype-to-phenotype correlations, is not always straightforward. This study presents a custom-designed multistep methodology to evaluate the impact of genetic variants found in patients with HL on protein function. This highlights the importance of developing combined molecular protein structure studies together with database analysis to evaluate and characterize the impact of reported and novel gene variants.

## Results

### Validation of variants and genetic diagnosis

Thirty-two patients with different forms of hearing loss were studied by WES, followed by data filtering based on 183 genes reported for this pathology. Approximately 90,000 variants were identified for each patient studied. Filtering reduced the list of candidate variants to 1–10 for each patient. After analysis following the American College of Medical Genetics and Genomics (ACMG) and Hearing Loss Variant Curation Expert Panel (HL-EP) parameters, variants were classified from benign to pathogenic in each patient and confirmed by Sanger sequencing and family segregation when available (full data are detailed in Supplementary Figure S1).

Overall, 27 different mutations were identified in the following 16 genes: *ACTG*1*, ADGRV*1 *(GPR*98*), CDH*23*, COL*4A3*, COL*4A5*, DFNA*5 *(GSDDE), EYA*4*, LARS*2*, LOXHD*1*, MITF, MYO*6*, MYO*7A*, TECTA, TMPRSS*3*, USH*2A and *WSF1* in 20 of the 32 studied patients (62.5%). The other 12 cases that remained undiagnosed had recessive variants in the heterozygous state or variants that failed to segregate with the pathology in the family. Therefore, since they did not fulfill the ACMG criteria, they were not reported as positive. Since neither large insertions/deletions nor repetitions or deep intronic variants were studied, exome data remain available for further analysis.

**Figure 1:**
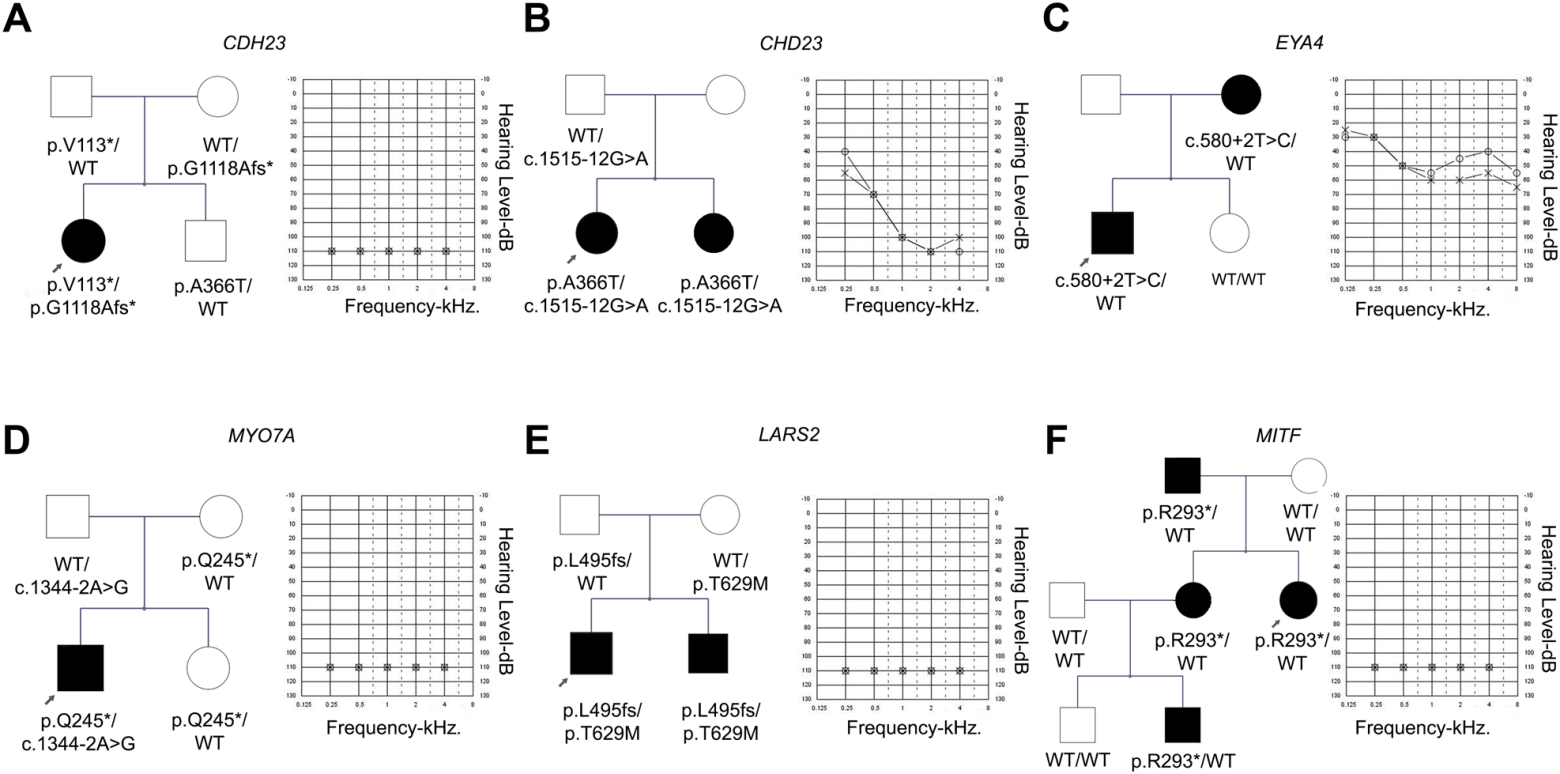
Pedigrees and audiograms of some of the families. All variants were identified by whole-exome sequencing and confirmed by Sanger sequencing. (**A**) Two novel variants in the *CDH*23 gene were identified, c.337del, p.(Val113*) and c.3353del, p.(Gly1118Alafs*7), in a patient with Usher signs. (**B**) Two previously reported variants were identified in *CDH*23: c.1515-12G > A, reclassified as likely pathogenic after manual curation, and c.1096 G > A, p.(Ala366Thr) classified as benign based on its high population frequency. (**C**) Postlingual bilateral moderate hearing loss caused by a novel heterozygous variant in *EYA*4: c.580+2T > C (splicing). (**D**) One-year-old boy with nonsyndromic isolated prelingual hearing loss and no retinal or vestibular pathologies at the time of study. Novel variants c.733C > T, p.(Gln245*) and c.1344-2A > G (splicing site mutation previously reported in ClinVar) in *MYO7*A were detected. (**E**) Two congenital bilateral profound cochlear implanted sisters with variants in *LARS*2: novel c.1481dup, p.(Leu495Thrfs*31*) and previously reported c.1886C > T, p.(Thr629Met). (**F**) Previously reported nonsense mutation c.877C > T, p.(Arg293*) in *MITF* cosegregated with pathology in four affected members of the family with nonsyndromic hearing loss.

In positive cases, segregation within the family was performed in 16 out of the 20 cases, and variant inheritance was established. All variants are listed in Table 2, together with the patients´ auditory phenotype (pedigrees are detailed in Fig. 1 and Supplementary Figure S1).

Among the 27 different mutations identified in 9 genes, 11 were novel, since they were not reported in deafness and Leiden Open Variation Database (LOVD) databases or in PubMed as related to pathology: c.12829C > T, p.(Arg4277*) in *ADGRV*1; c.337del, p.(Asp109*) and c.3352del, p.(Gly1118Alafs*7) in *CDH*23; c.3500G > A, p.(Gly1167Glu) in *COL*4A3; c.1183C > T, p.(Pro395Ser) and c.1759C > T, p.(Pro587Ser) in *COL*4A5; c.580+2T > C in *EYA*4; c.1481dup, p.(Leu495Profs*31) in *LARS*2; c.1939T > C, p.(Phe647Leu), in *MYO*6; c.733C > T, p.(Gln245*) in *MYO*7A and c.242C > G, p.(Ser81*) in *TMPRSS*3 genes*.* Values for each bioinformatic predictor, as well as its final interpretation using the Varsome platform manually adjusted, are detailed in Supplementary Table S4.

Five of the thirty-two patients under study presented features compatible with syndromic forms of hearing loss: 2 Alport, 2 Usher and 1 Waardenburg type 2 (WS2) (Table 1). Both Alport and Usher cases resulted in causative mutations in genes related to a syndromic phenotype, and the Waardenburg patient remained undiagnosed (nevertheless large deletions and/or insertions cannot be ruled out by this approach). Three additional cases presenting nonsyndromic HL at consultation resulted in pathogenic variants in genes associated both with syndromic and nonsyndromic forms: 2 Usher syndrome and 1 Perrault syndrome. These molecular genetic results might modify the future clinical outcome, genetic counseling and clinical follow-up to the patients and families. Of the 20 patients with identified variants, 13 required no further studies since they had nonsyndromic hearing loss.

**Table.1:** Reported phenotype characteristics of the 32 patients evaluated in this study.

Characteristic	Number
**Sex**	
Male	14
Female	18
No family history	16
Family History	16
Autosomal recessive	3
Autosomal dominant	13
**Onset**	
Congenital	16
Postlingual	16
**Physical exam**	
No other signs	27
Syndromic	5
Alport	2
Usher	2
Waardenburg	1

#### Clinical characteristics of relevant cases

##### Case #1

Two novel variants in *CDH*23 (NM_022124.5) were identified, c.337del, p.(Val113*) and c.3353del, p.(Gly1118Alafs*7), in a 23-year-old patient with cochlear implant in childhood (Fig. 1A). Both variants were associated with Usher Type 1D and resulted in truncated proteins, probably with no residual function. According to HL-EP recommendations, they were both interpreted as pathogenic. Both parents were healthy carriers by Sanger sequencing. The patient already showed some initial retinopathy compatible with Usher signs, further indicating that the mutations were causative of the phenotype and that the signs will progress.

##### Case #2

The patient had postlingual bilateral moderate hearing loss with a U-shaped audiogram, which was caused by a novel heterozygous variant in the *MYO*6 gene (NM_004999.4): c.1939T > C in exon 18 p.(Phe647Leu). The patient had four affected siblings (not available for molecular diagnosis). Thus, the variant was linked to a dominant form of inheritance (DFNA22). To further study the effect of the variant in the MYO6 structure, a model of the affected motor domain bearing the p.(Phe647Leu) mutation was generated (Fig. 3).

##### Case #3

The patient had postlingual bilateral moderate hearing loss that was caused by a novel heterozygous variant in *EYA*4: c.580+2T > C (splicing site). The variant cosegregated with the affected mother in the family, consistent with a dominant form of inheritance (Fig. 1C).

##### Case #4

Two variants in *MYO7*A (NM_000260.3) were identified: c.1344-2A > G (splicing site mutation reported in ClinVar for Usher Syndrome type I, either in homozygous or compound heterozygous state; rs111033415), in trans with the novel nonsense variant c.733C > T, p.(Gln245*). As this nonsense variant produces a stop codon, it will most likely result in a truncated protein. Thus, both of the variants meet the criteria to be classified as pathogenic for Usher syndrome type 1B or DFNB2 in an autosomal recessive manner. Segregation in the family was confirmed by Sanger sequencing. The affected proband, a 1-year-old boy, presented isolated prelingual hearing loss at the time of genetic diagnosis, with no retinal or vestibular pathologies (Fig. 1D). Since syndromic and nonsyndromic forms have been reported due to mutations in the *MYO*7A gene, patient clinical follow-up was recommended.

##### Case #5

**Figure 2:**
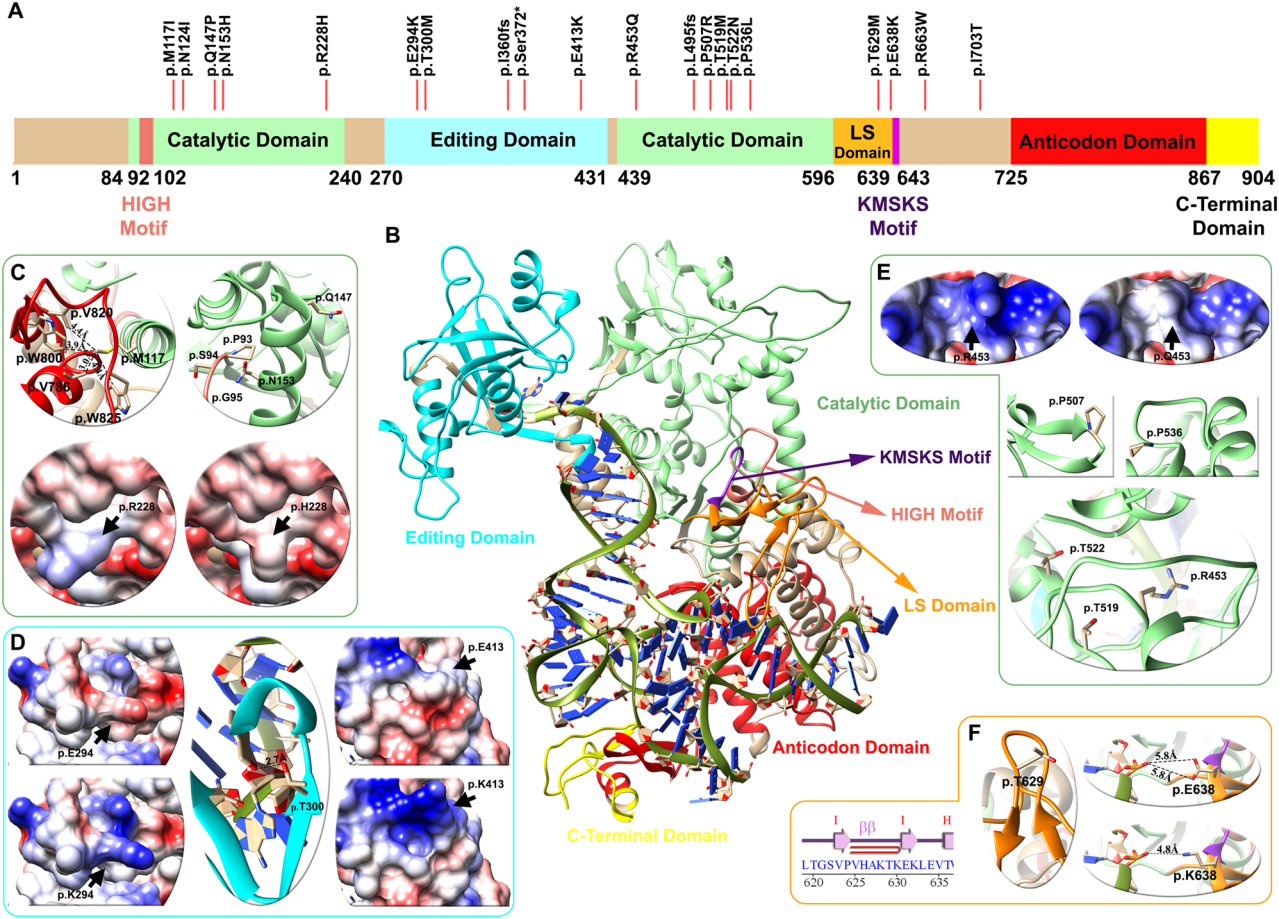
Domain architecture, mapping of variants and structural analysis of the LeuRS protein (*LARS2* gene). (**A**) Linear representation of the LeuRS protein with its domains and motifs: HIGH motif (pink), catalytic domain (light green), editing domain (cyan), LS domain (orange), KMSKS motif (purple), anticodon domain (red), and C-terminal domain (yellow). Red lines depict the location of the p.Thr629Met variant of case # 5 patient and 19 other pathogenic/likely pathogenic variants found in databases. (**B**) Human LeuRS molecular homology model, with the representation of domains and motifs. The zoom in shows variant analysis performed in three domains: light green for the catalytic (**C**,**E**), cyan for the editing (**D**) and orange for the leucine-specific (LS) (**F**) regions. For each domain mutations affect the electrostatic surface of the protein as well as the distance between neighboring residues. The p.Thr629Met variant lies in the LS domain between the hairpin of beta strand I–I, altering the folding of this loop and compromising the stability of the region (Zoom F). Detailed information regarding the genetic variants analyzed can be found in Table 3.

A familial case with two affected cochlear implanted siblings (8- and 12-year-old boys). Two variants were identified in *LARS*2 (NM_015340.3): c.1886C > T, p.Thr629Met was previously reported only once in ClinVar related to Perrault syndrome, and the novel c.1481dup, p.(Leu495Thrfs*31) (Fig. 1E). This latter novel frameshift mutation is predicted to be pathogenic based on its truncating effect on LeuRS, leading to the loss of its catalytic, leucine-specific and anticodon-binding domains. Pathogenicity was further confirmed by the deleterious effect of the mutation on the LeuRS structure through molecular modeling analysis (Fig. 2). Segregation analysis indicated that the parents were carriers for the mutations.

##### Case #7

A family case with a proband diagnosed with prelingual bilateral profound sensorineural hearing loss and three other affected members with a similar phenotype (son, sister and mother), who were cochlear implanted. The reported nonsense mutation c.877C > T (NM_000248.3) in *MITF* was found and predicted to be pathogenic, leading to an early truncated and nonfunctional protein p.(Arg293*). The variant cosegregated with the pathology in all of the affected members of the family. The same mutation has been reported in a family with Waardenburg syndrome type 2 (WS2)^15^. It is striking that no Waardenburg signs were observed in any of the members of the family (Fig. 1F).

##### *Case #8*:

An affected girl (15 years old) with isolated postlingual hearing loss and a sloping audiogram was diagnosed at 11 years old. Heterozygous variants were detected in *TMPRSS3* (NM_024022.2): c.1276 G > A, p.(Ala426Thr), reported several times as likely pathogenic in ClinVar supporting a deleterious effect (rs56264519) and the novel c.733C > T, p.(Ser81*) mutation. Parents were found to be carriers of these mutations, consistent with recessive inheritance.

##### Case #11

A 27-year-old patient with high frequency hearing loss. Two variants were identified in *CDH*23: c.1515-12G > A, previously reported as variant of uncertain significance (VUS) and now reclassified as likely pathogenic, and c.1096G > A, p.(Ala366Thr) classified as benign based on its high population frequency (BA1 applied) (Fig. 1B). It remains unclear whether these genetic findings are related to the pathology in this family, since the proband had no retinopathies or vestibular abnormalities, as seen in Usher Type D syndromes associated with mutations in *CDH*23 (OMIM #601067).

### Variant curation

After WES analysis, 28 different variants were found in 20 patients. Sixteen variants were already reported in the ClinVar database: 5 pathogenic, 6 pathogenic/likely pathogenic, 1 VUS, 3 conflicting interpretations and 1 benign (Table 2). We reanalyzed the 16 reported variants according to the Expert Panel specified recommendations for ACMG rules and/or manually adjusted them with evidence of segregation within the family and data from the literature. Eleven of these 16 variants changed their previous category (69%) (Table 2).

**Table.2:** Relevant Variants identified by WES.

ID	Gene (Transcript ID)	Genotype	Change	dbSNP	Phenotype of patient	Segregation (inheritance)	Reference	ClinVar report	After curation	Criteria applied
1	*CDH*23 (NM_022124.5)	c.337del	p.(Val113*)	–	Usher syndrome PL, PR, B, PF, CI	Maternal	This work	–	Pathogenic	PM2, PVS1, PM3, PP4
		c.3353del	p.(Gly1118Alafs*7)	–		Paternal	This work	–	Pathogenic	PM2, PVS1, PM3 and PP4
2	*MYO*6 (NM_004999.4)	c.1939T > C	p.(Phe647Leu)	rs752585373	PL, B, M	Non available	This work	–	VUS	PM2, PP3
3	*EYA*4 NG_011596.2 (NM_004100.5)	c.580+2T > C	splicing	–	PL, B, M	Maternal	This work	–	Pathogenic	PM2, PVS1, PP1_Sup
4	*MYO7*A NG_009086.2 (NM_000260.4)	c.733C > T	p.(Gln245*)	–	C, PL, B, PF, CI	Maternal	This work	–	Pathogenic	PVS1, PM2, PM3
		c.1344-2A > G	Splicing	rs111033415		Paternal	^65^	Pathogenic	Pathogenic	PM2, PVS1, PM3_S, PP4
5	*LARS*2 (NM_015340.3)	c.1481dup	p.(Leu495Thrfs*31)	rs762797278	C, B, PF, CI	Paternal	This work	–	Pathogenic	PVS1, PM2, PM3, PP1_Sup
		c.1886C > T	p.Thr629Met	rs398123036		Maternal	^16^	Pathogenic	Likely Pathogenic	PM2, PM3_S, PP1_Sup, PP4
6	*ADGRV*1 /*GPR*98 (NM_032119.3)	c.12829C > T	p.(Arg4277*)	–	Usher syndrome B, P, PR	Non available father	This work	–	Pathogenic	PVS1, PM2, PM3, PP1_Sup, PP4
		c.956dup	p.(Asn319Lysfs*6)	rs752179149		Maternal	^66^	Pathogenic	Pathogenic	PVS1, PM2, PM3, PP1_Sup, PP4
7	*MITF* (NM_000248.3)	c.877C > T	p.(Arg293*)	–	C, B, PF, CI	Segregation confirmed	^15^	–	Pathogenic	PM2, PVS1_S, PP1_S
8	*TMPRSS*3 (NM_024022.3)	c.1276G > A	p.Ala426Thr	rs56264519	PL, B. sloping audiometry	Maternal inheritance	^67^	Pathogenic/ Likely Path	Pathogenic	BS1_Sup, PM3_VS, PP1_S, PS3_Sup
		c.242C > G	p.(Ser81*)	rs757110501		Paternal inheritance	This work	–	Pathogenic	PVS1, PM2, PM3, PP1_Sup
9	*WFS*1 (NM_006005.3)	c.2590G > A	p.(Glu864Lys)	rs74315205	B, CI		^68^	Pathogenic/ Likely Path	Likely Pathogenic	PM2, PS4_M, PP1_Mod, PP3
10	*USH2*A NG_009497.2 (NM_206933.4)	c.1841-2A > G	Splicing	rs397518003	PR, B, M. No retinopathies	Maternal inheritance	^69^	Pathogenic	Pathogenic	PM2, PM3_VS,PP4 PP1_M, PS3_S
		c.10712C > T	p.(Thr3571Met)	rs202175091		Paternal inheritance	^70^	Pathogenic/ Likely Path	Pathogenic	PM2, PM3_VS, PP4, PP1_M
11	*CDH*23 NG_008835.1 (NM_022124.5)	c.1096G > A	p.(Ala366Thr)	rs143282422	B, High-frequency affected No retinopathies	Maternal	^71^	Benign	Benign	BA1
		c.1515-12G > A	splicing	rs369396703		Paternal	–	VUS (validated by HL–EP)	Likely Pathogenic	PM2_Sup, PM3, PP1_Sup, PP3, PP4
12	*COL*4*A*3 (NM_000091.5)	c.3500G > A	p.(Gly1167Glu)	–	Alport Syndrome. Hematuria	De novo (maternal)	This work	–	Pathogenic	PM2, PS2, PM1, PM5, PP3
		c.4649T > G	p.(Val1550Gly)	rs200655479			^72^	Conflicting Interpretation (VUS/LP)	VUS	PM2_Sup, PP3
13	*DFNA*5 (NM_004403.2)	c.119dup	p.(Lys41Glufs*113)	rs758488919	No familial history	De novo	–	Conflicting Interpretation (VUS/LB)	Benign	BA1, PS2
14	*COL*4*A*5 (NM_000495.3)	c.1183C > T	p.(Pro395Ser)	–	C, B, S		This work	–	VUS	PM2
15	*COL*4*A*5 (NM_000495.3)	c.1759C > T	p.(Pro587Ser)	–	PL, PR, B, M		This work	–	VUS	PM2, PP3
16	*COL*4*A*5 (NM_000495.3)	c.3659G > A	p.(Gly1220Asp)	rs104886251	Alport Syndrome. Hematuria		^73^	Pathogenic	Pathogenic	PM2, PM1, PP3, PP4, PS4_Sup
17	*WFS*1 (NM_006005.3)	c.2327A > T	p.(Glu776Val)	rs56002719	B, M, PR. High frequencies	Maternal inheritance	^74^	Conflicting Interpretation (VUS/B/LB)	Benign	BA1, PP3, PP1_Sup, BS4
18	*TECTA* (NM_005422.4)	c.5668C > T	p.(Arg1890Cys)	rs121909063	PL, M-S	Segregation confirmed. Paternal Inheritance	^75^	Likely pathogenic	Pathogenic	PM2, PP1_VS, PS4_Sup
19	*LOXHD*1 (NM_144612.6)	c.4480C > T (homozygous)	p.(Arg1494*)	rs201587138	C, B, PF	Segregation confirmed	^76^	Pathogenic/ Likely Path	Pathogenic	PVS1, BS1_Sup, PM3_S, PP1_M
20	*ACTG1* (NM_001614.5)	c.353A > T	p.(Lys118Met)	rs104894544	PL, B, M-S	Segregation confirmed. Paternal Inheritance	^77^	Likely pathogenic	Pathogenic	PS4_Sup, PM2, PP5, PP1_S,PP3

All variants were curated following the Hearing Loss Expert Panel recommendations. The phenotype of the patients is indicated as follows: *C* congenital, *PL* postlingual, *PR* progressive, *B* bilateral, *M* moderate, *PF* profound, *S* severe, *CI* cochlear implanted.

For instance, one variant changed from pathogenic to likely pathogenic, [p.(Thr629Met) in *LARS*2], two from likely pathogenic to pathogenic [p.(Arg1890Cys) in *TECTA*] and [p.(Lys118Met) in *ACTG*1], three from likely pathogenic/pathogenic to pathogenic [p.Ala426Thr in *TMPRSS*3, p.(Thr3571Met) in *USH*2A, p.(Arg1494*) in *LOXHD*1] and one from likely pathogenic/pathogenic to likely pathogenic [p.(Glu864Lys in *WFS*1]. Interestingly, two variants changed from conflicting interpretation to benign after reanalysis [p.(Lys41Glufs*113) in *DFNA*5 and p.(Glu776Val) in *WFS*1], and another changed to VUS [p.(Val1550Gly) in *COL4A*3]. Variant c.1515-12G > A in *CDH*23 reported as VUS in the ClinVar database was reclassified as likely pathogenic, reinforcing its causal relationship with the pathology.

In the case of novel variants, classification was established following the standard protocol as per ACMG/AMP for variant interpretation for genetic hearing loss and the updated recommendations of the ClinGen Hearing Loss Expert Panel, and in some cases (*LARS*2 and *MYO*6), their final classification was further established through molecular modeling and in silico strategies.

### Combined in silico analysis

Functional assays are essential for the interpretation of missense variants associated with pathology. However, experiments for functional validation are time consuming and not always feasible in the clinical context. Therefore, bioinformatic tools that predict protein malfunction appear to be valid predictable tools of pathogenicity. We implemented full modeling and domain modeling as bioinformatic approaches to determine the in silico implications of missense variants.

#### Full modeling of LeuRS (*LARS*2 gene)

The *LARS*2 gene encodes a mitochondrial leucyl-tRNA synthetase (LeuRS) that catalyzes the aminoacylation of a specific tRNA. The protein architecture of LeuRS includes motifs that are catalytically important (HIGH and KMSKS) and different domains: catalytic, editing, leucine-specific (LS), anticodon-recognition, and the C-terminal domains (C-ter) (Fig. 2). Sequence variants in *LARS*2 have been previously associated with Perrault syndrome, characterized by premature ovarian failure, hearing loss and other severe multisystem metabolic disorders (OMIM #604544).

In Case #9, we identified two variants: c.1481dup, p.(Leu495Thrfs*31) and c.1886C > T (p.Thr629Met). The former is novel and predicted to yield a truncated nonfunctional protein. Although the latter variant has already been reported, we performed a deeper follow-up analysis to understand the impact of the mutation on the translated protein. Thus, we conducted molecular modeling of the entire human LeuRS and analyzed its stability, electrostatic surface and tRNA interaction. In addition to the p.Thr629Met variant found in the proband of Case #5, we included in our analysis 17 additional missense variants reported as likely pathogenic or pathogenic for the *LARS2* gene in LOVD, Deafness Variation Database and ClinVar (2 frameshift and 1 nonsense reported variant were not included). These variants are identified above the primary structure of LeuRS (Fig. 2A). Most of the variants were located in the catalytic, LS and editing domains, and none were located in the C-terminal or anticodon domain.

The model shows that protein stability was altered in 7 of the variants analyzed (41%), the electrostatic charge in 3 (18%) and the tRNA-protein interaction in 3 (18%) of the previously reported mutations. The analysis was nonconclusive for 4 variants (24%) (Table 3).

**Table.3:** Evaluation of genetic variants in LeuRS.

Variant and amino acid change (NM_015340.3)	Effect †	Stability ^+^	ClinVar	Deafness Variation Database/LOVD	Hearing loss expert panel classification + Modeling	Reference
c.351G > C; p.(Met117Ile)	Stability	4.11 ± 0.63	–	P/LP	Likely Pathogenic (PM2, PM3, PP1, PP4,PP3)	PMID: 26,970,254
c.371A > T; p.(Asn124Ile)	Non conclusive	0.41 ± 0.66	P	P/–	Likely Pathogenic (PM2, PM3_Strong, PP4)	PMID: 28,708,303
c.440A > C; p.(Gln147Pro)	Stability	1.49 ± 0.12	LP	LP/–	Likely Pathogenic (PM2, PM3, PP4, BP4_Supporting, PP3)	SCV000994657.1
c.457A > C; p.(Asn153His)	Stability	3.36 ± 0.82	LP	LP/–	Likely Pathogenic (PM2, PM3 PP3, PP4)	PMID: 32,423,379
c.683G > A p.(Arg228His)	Electrostatic Surface	0.40 ± 0.02	LP	LP/LP	Likely Pathogenic (PM2, PM3_Supporting, PP3, PP4)	PMID: 28,000,701
c.880G > A; p.(Glu294Lys)	Electrostatic surface	0.67 ± 0.14	–	P/LP	Likely Pathogenic (PM2, PM3_Strong, PP4, PP3)	PMID: 28,000,701; 3,276,773; 29,205,794
c.899C > T; p.(Thr300Met)	tRNA interaction	0.12 ± 0.84	P	P/P	Likely Pathogenic (PM2, PM3, PP1, PP3, PP4)	PMID: 26,657,938
c.1077del; p.Ile360fs	LoF	–	P	P/P	Pathogenic (PVS1, PS3_Supporting, PM2, PM3, PP4)	PMID: 23,541,342
c.1115C > G; p.(Ser372*)	LoF	–	LP	LP/–	Pathogenic (PVS1, PM2, PP4)	SCV000891207.1
c.1237G > A; p.(Glu413Lys)	Electrostatic surface	0.14 ± 0.02	LP	LP/–	VUS (PM2, BP4, PP4)	SCV001244305.1
c.1358G > A; p.(Arg453Gln)	Electrostatic surface/Stability?	1.42 ± 0.3	–	P/P	Likely Pathogenic (PM2_Supporting, PM3, PP3, PP4)	PMID: 27,650,058
c.1481dup; p.(Leu495fs)	LoF	–	–	P/–	Pathogenic (PVS1, PM2, PM3, PP1_Supporting)	This study paper
c.1520C > G; p.(Pro507Arg)	Stability	2.20 ± 0.07	LP	LP/–	Likely Pathogenic (PM2, PM3, PP1_Supporting, PP3)	SCV000731430.1
c.1556C > T; p.(Thr519Met)	&	− 0.44 ± 0.25	–	P/–	Likely Pathogenic (PM2, PM3, PP1_Supporting, PP3, PP4)	PMID: 29,205,794
c.1565C > A; p.Thr522Asn	&	− 0.59 ± 0.08	LP	P/LP	Likely Pathogenic (PM2_Supporting, PM3_Strong, PS3_Supporting, PP3, PP4)	PMID: 23,541,342
c.1607C > T; p.(Pro536Leu)	Stability	6.82 ± 1.87	LP	LP/–	Likely Pathogenic (PM2, PS3_Supporting, PM3, PP3, PP4)	Accession: SCV000994658.1
c.1886C > T; p.Thr629Met	Stability	2.56 ± 0.19	P	P/P	Likely Pathogenic (PM2, PM3_Strong, PP1_Sup, PP4)	PMID: 23,541,342
c.1912G > A; p.(Glu638Lys)	tRNA interaction	− 0.06 ± 0.50	P	P/P	Likely Pathogenic (PM2, PM3, PP1_Supporting, PP3, PP4)	PMID: 26,657,938
c.1987C > T; p.(Arg663Trp)	Stability	2.94 ± 0.32	P	P/–	Likely Pathogenic (PM2, PS3_Supporting, PM3_Strong, PP3, PP4)	PMID: 28,708,303
c.2108T > C; p.(Ile703Thr)	tRNA interaction	0.67 ± 0.11	–	LP/–	Likely Pathogenic (PM2, PM3, PP3, PP4)	PMID: 32,767,731

^†^Classification of variants according to structural criteria. + ΔΔG Energy evaluation for pathogenic and likely pathogenic genetic variants, FOLDX: |X|± SD (n = 5). *LoF* loss of function, *P* pathogenic, *LP*: likely pathogenic. This information was compiled from the LOVD3, ClinVar and deafness variation databases until 26 January 2021. &: both residues are oriented facing the core, causing a probable steric effect. The new model of LeuRS was considered a new parameter (PP3 score applied) to classify the variants reported in databases according to the Hearing Loss Expert Panel classification.

The p.Thr629Met variant detected in the patient of Case #5 lies in the LS domain between the hairpin of beta strand I–I altering the folding of this loop, compromising the stability of the region (Fig. 2F and Table 3). An additional previously reported variant, p.(Glu638Lys), also lies in the LS domain and alters the interaction with de t-RNA through the change of a negative to a positive side chain of the residue (Fig. 2F). Ten genetic variants are located in the catalytic domain of the protein. According to the model, p.Met117 (light green helix in Fig. 2C) interacts with residues p.Trp800, p.Val820, p.Trp825 and p.Val786, of the anticodon domain (red helix in Fig. 2C). The methionine to isoleucine change in p.(Met117Ile) results in altered protein stability (Table 3), as do p.Gln147Pro and p.Asn153His variants found in the H4 α-Helix. In addition, the p.(Asn153His) change affects protein stability, particularly the interaction between p.Pro93, p.Ser94 and p.Gly95 of the HIGH motif. The variant p.(Arg228His) abolishes a negative charge, altering the protein electrostatic surface (bottom Fig. 2C).

Under the magnifying glass of the present model, variant p.Arg453Gln lies within the catalytic domain of the protein, in contrast with the previously reported model that positioned it in the t-RNA binding domain ^16^. Thus, the pathogenicity of p.(Arg453Gln) is related to a combination of a change in surface electrostatic charge and a steric effect of this residue in the region (Table 3 and Fig. 2E). Mutations p.(Thr519Met) and p.Thr522Asn affect the structure of the catalytic domain through a steric effect. In addition, p.(Pro507Arg) affects the structure of the loop between the G-G β-strand, and p.(Pro536Leu) affects the structure of the H19-H20 α-helix (Fig. 2E, green).

Three missense variants were found in the editing domain. Variants p.(Glu294Lys) and p.(Glu413Lys) generate a change from glutamic acid to arginine, decreasing the electrostatic surface charge. The p.Thr300 residue, mutated to M in a previously reported patient^17^, is crucial for leucine-tRNA edition in the β-strand of this domain, as observed in the center of Fig. 2D.

The new model of LeuRS presented in the present work can be considered a new parameter (PP3 score applied) to classify variants reported in databases according to the Hearing Loss Expert Panel classification. The final classification of reported *LARS2* variants is detailed in Table 3.

#### Modeling of the N-terminal motor (head) domain of the MYO6 protein

Myosins are actin-based motor molecules with ATPase activity. Myosin VI is a reverse-direction motor protein that moves toward the minus-end of actin filaments and plays a crucial role in the organization of the stereociliary bundle and the maintenance of the cuticular plate anchoring of stereocilia rootlets in hair cells^18^. Mutation p.(Phe647Leu) in *MYO*6 detected in Case #6 was not previously reported in ClinVar and was not found in the GnomAd (Genome Aggregation Database) or 1000 Genomes databases. To determine the potential effect of the missense variant on MYO6 function, we further performed in silico studies. The bioinformatic analysis predicted that c.1939T > C, (p.Phe647Leu) is a disease causing variant by Mutation Taster (0,81), damaging by PolyPhen-2 HumDiv (score 0.986), damaging by SIFT (0), deleterious by CADD, damaging by PROVEAN (-5,7) and pathogenic by the UMD-Predictor (score 75), REVEL (0,9).

**Figure 3:**
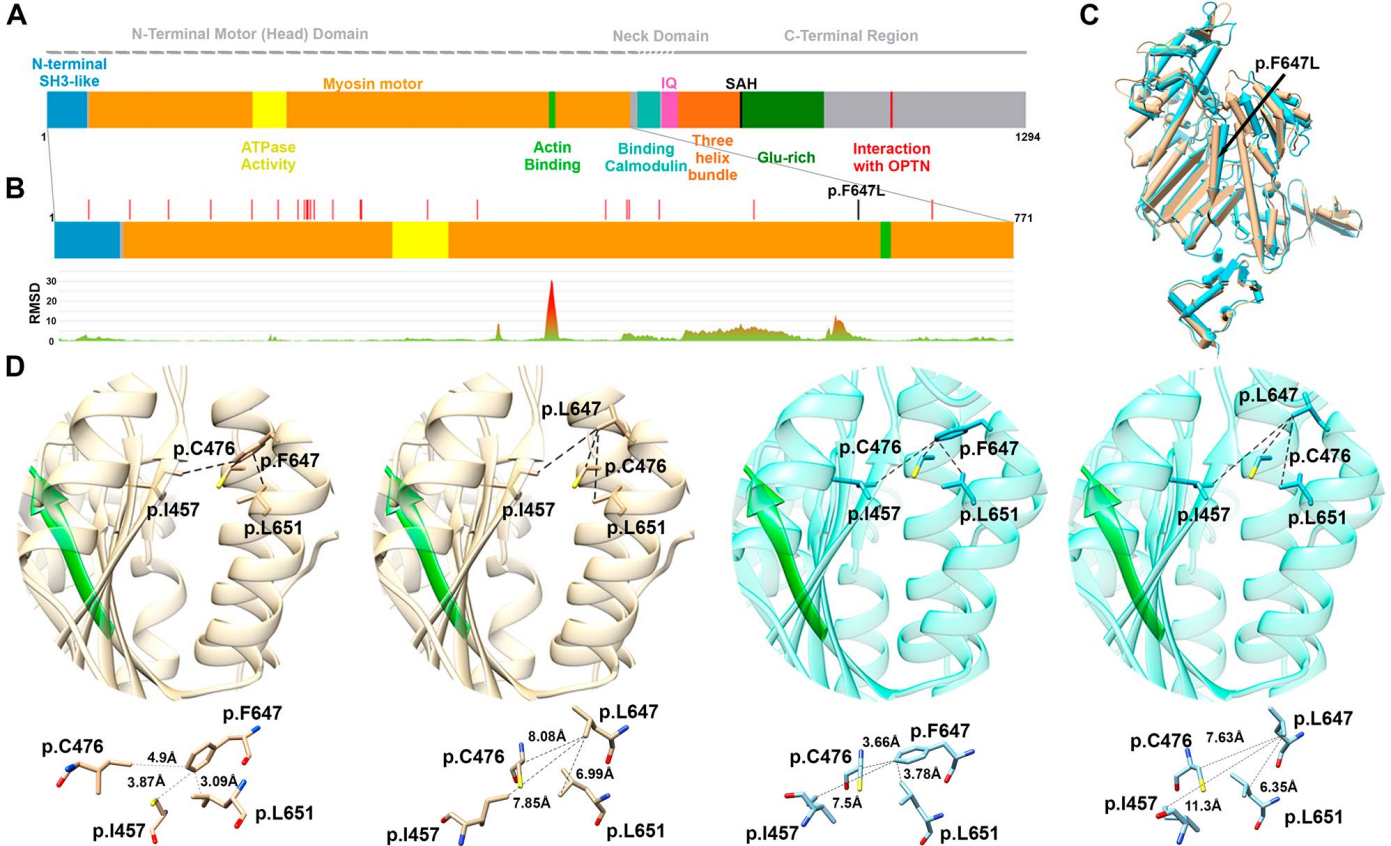
Motor head domain analysis of MYO6 protein. (**A**) Ideogram of MYO6. (**B**) RSMD analysis of the motor head domain shows peaks with high RMSD values, revealing that two areas (one including the p.(F647L)) are involved in regions with great motility between pre-powerstroke and Pi release conformations. (**C**) Motor head domain modeling. Pre-powerstroke conformation in light blue and Pi release in brown. The p.(Phe647Leu) is shown with a black arrow. (**D**) Zoom in of the alpha-helix at the 647 region. The interaction of the wild-type Phe647 residue or the mutated version Leu647 with the three nearest residues is shown for each protein conformation (Pi release in brown and pre-powerstroke in light blue). The β-strand in green represents the actin binding region. Distances in Å are detailed under each bubble, showing the increase in the distances for the mutated Leu647 residue in both conformations.

With the aim of further analyzing the impact of the mutation on the protein, specific protein domain modeling was performed in two conformations. The root mean-square deviation (RMSD) values of the protein backbone distances between the two MYO6 motor domain conformations (pre-powerstroke and PI release) were first calculated to determine whether the residue is located in a motil region. In particular, two high RMSD values were obtained for amino acid positions 395 to 405 and 621 to 649 in the two conformations. Therefore, amino acid changes in these regions could affect the interaction with nearby residues, affecting the proper function of the protein (Fig. 3B). Our analysis indicates that residue Phe647 is indeed located in a motile region between both conformations, resulting in a significant influence on the structure of the protein. This change leads to a steric effect in the amino acid side chain, altering interactions with nearby nonpolar residues. In particular, when Phe647 is mutated to Leu, the distance between the side chains of the three nearest residues (less than 5 Å in the wild type), Ile457, Cys476 and Leu651, is significantly increased. For the pre-powerstroke state conformation, the average distance between residue 647 and these amino acids is 4.98 Å (Phe) and 8.42 Å (Leu). For the Pi release state conformation, the average is 3.95 Å (Phe) and 7.64 Å (Leu). Thus, in both the pre-powerstroke and Pi release conformations, the average distance was almost twofold increased in the Leu647 variant (distances are detailed in Fig. 3D). In addition, model analysis shows that the alpha helix containing the Phe647 residue is adjacent to the actin binding region (Fig. 3C, in green β-strandand). Hence, it is possible to infer that alterations in the surrounding areas may affect the structure and/or function of the actin binding site.

## Discussion

Genotype–phenotype characterization in HL patients is not a straightforward endeavor. Nonequivocal genotypic information is crucial for the clinical care and genetic counseling of HI patients. Gene variants leading to frameshift and nonsense mutations or affecting canonical splice sites most likely lead to a null translation of the mutated allele. However, predicting the effect of DNA substitutions leading to missense mutations is far more complicated. These can lead to a myriad of effects that end in protein malfunction, including altered stability, nonfunctional protein domains and lack of catalytic function. Compiled by field experts gathered in the Hearing Loss Expert Panel, genotype validation is accelerating. In the present work, we report new gene variants in a cohort of Argentinian HI patients. These were curated following the ACMG and Hearing Loss Expert Panel guidelines. Moreover, the pathogenicity of some of the variants was validated through in silico protein analysis. In addition, the pathogenicity of previously reported variants in the ClinVar database was reclassified. The present work adds to the standardization of HI variant interpretation as a crucial step to provide consistent and accurate diagnoses for families and professionals involved, as well as for a better understanding of the mechanisms underlying disease pathogenesis.

Overall, 27 different mutations were identified in 16 hearing loss genes in 20 out of the 32 patients studied. The rate of genetic diagnosis was 63%, significantly higher than the 36% standard of care (*GJB*2/6 sequencing only), previously reported in our laboratory^8,19,20^. There was a significant diversity in the overall diagnostic rate. In patients with a family history, the diagnostic rate was 50% (8/16), while in isolated cases, 69% were diagnosed (11/16). This bias can be attributed to the fact that the isolated patients who were diagnosed had mostly syndromic forms, where the genetic target to be studied is more enclosed and has a higher diagnostic success rate. The curation procedure was effective since eleven of the sixteen (69%) reported variants changed or refined their category previously reported in ClinVar. In this regard, rigorous variant manual curation demonstrates its importance in accurate variant interpretation and hence precise genetic counseling to patients. These outcomes are in accordance with our previous report^19^.

The two sisters of Case #11 with nonsyndromic hearing loss exhibited two variants in *CDH*23, a gene that encodes a putative cell adhesion protein with multiple cadherin-like domains. *CDH*23 is responsible for both Usher syndrome and DFNB12 nonsyndromic deafness^1^. It has been established that nonsense, splice-site, or frameshift mutations in *CHD*23 are related to Usher 1D, whereas missense mutations are related to a milder phenotype (nonsyndromic HL, DFNB12). Moreover, concerning the type of hearing loss, previous genotype–phenotype reports showed that the majority of patients have some residual hearing at lower frequencies and a characteristic high frequency hearing loss sloping pattern. This is in accordance with the audiogram of our studied siblings^21,22^. After the curation process, variant c.1515-12G > A in *CDH*23, applying criteria PM2_Supporting, PP3, PM3, PP4 and PP1_Supporting, changed its previous classification in ClinVar from VUS to Likely Pathogenic. Further functional analysis, as well as new reports in Usher patients would provide confident evidence concerning its pathogenicity. Variant c.1096G > A; p.(Ala366Thr) was classified as benign due to its high population frequency in the Ashkenazi population (BA1 criteria applied). Hidden variants in gene regions that we did not explore, such as deep intronic mutations that can disrupt transcription regulatory motifs and/or noncoding RNA regions, might underlie the observed phenotype^22,23^. Taking into account all the evidence presented we cannot unequivocally conclude that these two variants are the cause of the hearing loss in this family. Nevertheless, due to the reclassification of c.1515-12G > A to likely pathogenic, the case is worth mentioning and discussed.

In Case #10 a nonsense variant was detected in *MITF*. Most mutations in *MITF* have been mostly associated with Waardenburg syndrome type 2, a dominant syndromic form of hearing loss. It is associated with hypopigmentation of the skin, hair and eyes, since *MITF* has a regulatory effect on *TYR* transcription, a key enzyme involved in melanin synthesis^24^. The variant identified in our patients had already been reported in a Waardenburg type 2 family case^15^. Notably, none of the four affected members of this family presented any other signs in addition to profound HL. This finding is in accordance with some reports in which variants in the *MITF* gene cause only hearing loss^25,26^. The variable phenotype expression could be explained by the presence of modifier genes, as well as interactions with environmental factors^27,28^.

Several bioinformatic algorithms have been developed to predict the functional consequences of single nucleotide variants in protein coding regions. These in silico approaches are an alternative to tedious and time-consuming experimental approaches to infer pathogenicity. Thus, the new model presented in the present work for the LeuRS human protein aided in defining the plausible pathogenicity of the identified p.Thr629Met variant in Case #9. Moreover, it can be further used to predict the pathogenicity of other reported variants. The p.Thr629Met mutation in the patient lies in the beta hairpin of LeuRS between strands I-I of the LS domains and leads to the disruption of the motif and protein stability. This is in accordance with the observation that beta hairpin motifs are implicated in protein stability^29,30^. Moreover, the substitution of p.Ala508 in Escherichia coli LeuRS (analogous to human LeuRS p.Thr629) with a nonpolar methionine disrupts the structure and/or position of the leucine-specific domain and thus shifts the location of the KMSKS loop and reduces the catalytic efficiency^16,31^. It should be noted that the present model of human LeuRS is an improved version of those previously reported^16,32^ since we used a variety of selected crystals. In this regard, the present model shows that most of the variants in the catalytic domain produce a severe effect on protein stability, affecting the proper function of the protein. Our new model shows that variants reported as pathogenic in databases effectively induce significant structural changes of the protein that would cause potential functional changes, playing an important role in genotype/phenotype prediction. Thus, we propose that this model should be used for future in silico predictions of variant pathogenicity in patients with Perrault syndrome, becoming a bridge between genomics and structural data to guide the interpretation of human genetic variants.

In Case #6, we identified a novel *MYO*6 variant c.1939T > C, p.(Phe647Leu) in a family with late-onset autosomal dominant nonsyndromic hearing loss. Most MYO6-known genetic variants present progressive hearing loss^33–35^ and myosin VI is required for structural integrity and proper functioning of inner ear hair cells^18,36^. More than half of the known pathogenic mutations are located in the motor head domain, indicating that it plays an important role in the function of MYO6^34^. The motor-head domain modeling approach applied in the present work reveals that mutation p.(Phe647Leu) alters the proper function, most likely through the increased distance of this amino acid from the three nearby nonpolar residues. Moreover, the structural change caused by the mutation in the alpha-helix of this highly motile region could potentially affect the contiguous actin binding region. In this regard, mutations in the MYO6 motor domain alter anchoring of the membrane of stereocilia to actin filaments, leading to disruption of hair bundle organization^36,37^.

In conclusion, the present work highlights the importance of the curation of genetic variants leading to HL following recommendations of experts for the correct phenotype-genotype correlation. Moreover, we show the importance of the incorporation of integrated workflows for predicting the biomedical impact of the variations identified by exome analysis. Most importantly, we propose a multitarget approach including genomics, protein structure and data analysis to guide the interpretation and standardization of human genetic variants leading to hearing loss.

## Materials and methods

### Subjects and selection criteria

Thirty-two unrelated Argentinean families were included in this study (Fig. 1 and Supplementary Figure S1). Hearing loss was bilateral and moderate to severe (45–95 dB) or profound (> 95 dB), and onset was either congenital or postlingual progressive (average 20–40 years). Audiological evaluation included pure-tone audiometry at four frequencies (0.5, 1, 2, and 4 kHz). The pure-tone average (PTA) was calculated from the audiometric thresholds. The HL patients were divided into three groups based on severity: moderate (41–70 dB HL), severe (71–95 dB HL), and profound (> 95 dB HL). The audiometric configurations were classified into low-frequency, middle-frequency (U-shaped), high-frequency and flat types^38^. Patients underwent auditory brainstem response (ABR), tympanometry, fundus examination, and cardiac and renal ultrasonography to detect undiagnosed syndromic forms*.* Clinical examinations revealed symptoms suggesting a syndromic form of deafness in five patients: two with visual defects, two others with hematuria, and one with hair pigment abnormalities. All data were reviewed by a clinical geneticist. The study was conducted in accordance with the Declaration of Helsinki, and the protocol was approved by the Ethics Committee of “Administración Nacional de Laboratorios e Institutos de Salud” (ANLIS-19122018) and “Fundación para la Lucha contra las Enfermedades Neurológicas de la Infancia” (FLENI -04,092,020). Written informed consent for testing and publication was obtained from patients or parents in the case of minors.

Genomic DNA was extracted from peripheral blood samples using the standard method. Quality and concentration were measured by agarose gel electrophoresis and absorbance-based nucleic acid quantification (Thermo Scientific–NanoDrop™). A total of 695 patients with different forms of deafness were recruited for *GJB*2/*GJB*6 mutation screening, identifying biallelic pathogenic mutations in 103 of them (15%). Thirty-two patients undiagnosed for *GJB*2/*GJB*6 mutations were selected for WES screening. The age of the patients varied between 6 months and 50 years. Seventeen out of the 32 patients (53%) were females, and fifteen (47%) were males. Among the 32 probands, 50% (16/32) were sporadic and 50% (16/32) had at least two affected relatives with HL (familial cases).

### Whole exome sequencing and bioinformatics analysis

Massive parallel sequencing was carried out on an *Illumina NovaSeq6000.* Base calling, read mapping and annotation of variants were performed by Macrogen Genome Sequencing Services (Macrogen, Korea) and data were processed according to the Genome-Analysis-Toolkit (GATK) best practices workflow. Variants for 183 deafness-related genes were filtered from the WES on samples from affected patients and relatives when available (analyzed genes are listed in Supplementary Table S1). The average read length covered 148 bp, with an average exon depth coverage in analyzed genes of 100X; 97% of targeted reads had > tenfold coverage. Aligned reads were compared to the human reference genome (*GRCh37/hg19*). After variant annotation, mutations that arose from known deafness genes were selected by filtering with an in silico panel using a homemade Python script pipeline. The missense, nonsense, insertion/deletion and splicing variants were selected from the detected variants. The minor allele frequency threshold (MAF) considered was ≤ 0.01 and 0.005 for recessive and dominant alleles, respectively, when compared with those reported in gnomAD—http://gnomad.broadinstitute.org/) and 1000 genomes (https://www.internationalgenome.org/1000-genomes-browsers/). The pathogenic potential of selected variants was analyzed using bioinformatic programs and databases: Mutation Taster—http://www.mutationtasetr.org^39^, PolyPhen-2—http://genetics.bwh.harvard.edu/pph/^40^, CADD (Combined Annotation Dependent Depletion)—http://cadd.gs.washington.edu/, UMD-Predictor—http://umd-predictor.eu.^41^, ClinVar—https://www.ncbi.nlm.nih.gov/clinvar/, LOVD—https://www.lovd.nl/, The Human Gene Mutation Database—http://www.hgmd.cf.ac.uk/, dbNSFP—https://sites.google.com/site/jpopgen/dbNSFP. All information was compiled and criteria rules were combined to reach a variant classification according to data retrieved from InterVar -http://wintervar.wglab.org/, Varsome—https://varsome.com/ and REVEL—https://sites.google.com/site/revelgenomics^42^. Once annotation of variants was performed, the number of significant variations was reduced from more than eighty thousand to a small, manageable number (1 to 10) of putative candidate disease-causative mutations for further validation. This process included various parameters, such as mode of inheritance, mutation localization, mutation type (nonsynonymous variants, splice acceptor or donor site mutations, and coding noninframe In/Dels), frequency, pathogenicity of variants, published reports, and so forth. Pathogenicity prediction and variant classification were assessed taking into account criteria defined by the ACMG and further modifications, as well as recommendations of the HL-EP and data from deafness variation databases (http://deafnessvariationdatabase.org/)^43–45^. Variants were classified as pathogenic, likely pathogenic, VUS, likely benign or benign. Patients were informed of the results, and clinical follow-up was recommended when necessary, since some likely pathogenic variants were identified in syndromic genes, before the onset of additional clinical manifestations. Positive results were always confirmed by Sanger sequencing prior to reporting, and segregation through family was performed when possible.

### Variant curation

To further analyze and validate the identified variants in patients, a combined in silico analysis was performed. In the first curation step, the prediction of pathogenicity of the variants previously reported in ClinVar was reanalyzed following the guidelines mentioned before^43^, collecting new data regarding segregation, new PubMed reports or publications and new updated data of frequency and phenotype/genotype correlations. The putative pathogenic effect of novel variants was manually scored using Varsome^46^.

### Bioinformatic analysis

To predict the potential consequences of some of the missense variants on protein function, different bioinformatic analyses were performed. These included a molecular homology modeling (MHM) approach and structural analysis of the mutated proteins. The line protein graphs in figures were generated with the PyGame library in the Python 2.7 programming language (http://www.python.org)^47^.

#### Molecular modeling and structural analysis

MHM was performed for mitochondrial (LeuRS) (UniProt_ID Q15031-1) and the MYO6 motor-head domain (UniProt_ID Q9UM54-3, between amino acid residues 4 and 771). The evaluation criteria to select the template for the MHM were as follows: (1) protein sequence identity as template, (2) X-ray crystal resolution less than 2.4 Å, (3) crystallography conditions, (4) chain length and amount of residues of each template with respect to sequence identity and gaps, (5) structural conformation of each protein template, and (6) species of the template. The alignment between template and target sequences was performed with structural alignments (Expresso extension) in the T-Coffee web server^48,49^, MODELLER alignment scripts and Kluskal-Wallis in MEGA5 software, taking into account the secondary structures and topology of the regions^50^. Homology modeling was generated using the MODELLER program^51^. The models were first optimized with the variable target function method with conjugate gradients and then refined using molecular dynamics with simulated annealing^52^. Model quality evaluation was performed using DOPE^53^, QMEAN/QMEANDisCo^54,55^ and ProSA^56^ to predict local pairwise residue-residue distances to the assessed model. The UCSF Chimera program^57^ and the backbone-dependent rotamer library were used for structural interpretation and visualization^58^.

For the LeuRS protein model, template structures were selected from the RCSB Protein Data Base^59^ and UniProt (https://www.uniprot.org/). Fifteen potential templates were considered using a cutoff in sequence identity larger than 36% and a resolution less than 2.5 Å. For the alignment between templates and the LeuRS sequence, the N-terminal mitochondrial targeting signals (presequences) were deleted using MitoFates^60^. Structural regions that did not have reference templates were not considered for the structural analysis and are not shown in the visualization of the structure. After evaluation, six structures in the editing conformation were selected (Supplementary Table S2). Thirty molecular homology models with the selected templates were generated, and the final structure of the tRNA was determined with the 4AS1 template.

The MYO6 protein structure is divided into three regions: an N-terminal motor-head-domain (which comprises the N-terminal SH3-like, myosin motor, actin binding site and ATP catalytic region), followed by a neck domain (with a calmodulin-binding linker domain and a single IQ motif), and a C-terminal tail region (with a three-helix bundle region, an SAH domain and a unique globular domain required for interaction with other proteins such as cargo-binding)^61^ (Fig. 3A). The modeled N-terminal motor (head) contains the N-terminal SH3-like (aa 4–53) and myosin motor (aa 57–771) domains with 98.15% identity between the target sequence and templates and a resolution of less than 2.5 Å. Modeling was performed in two MYO6 conformations according to ATPase cycle states: the pre-powerstroke, which is the key force-generating step (PDBID: 2V26, 4DBR, 4E7Z), and the next state with the release of phosphate, Pi release (PDBID: 4PFP, 4PFO, 4PJN)^62^ (Supplementary Table S3).

##### Stability analysis

To evaluate the effect of mutations on the stability/folding of LeuRS, we first analyzed the structural and energetic details of the interactions for each mutated residue using FoldX (http://foldxsuite.crg.eu/).^63^. Structures were optimized to the FoldX force field command from the molecular homology models. The ∆∆G values were estimated as the difference between the energy of the wild-type protein and the average of five replicas for each point mutation^64^. Values above 1,6 kcal/mol (twice the standard deviation) were considered to significantly destabilize the protein. To favor the wild-type conformation, all residues involved in tRNA interactions (5 Å distance) were fixed when optimizing the structures to the FoldX force field.

## Supplementary Information

Supplementary Information 1.

Supplementary Information 2.

Supplementary Information 3.

Supplementary Information 4.

Supplementary Information 5.

## Data Availability

The genetic variant data that support the findings of this study are openly available in ClinVar at [http://www.clinvar.com/], reference number SUB10104745. New LeuRS and MYO6 protein models are openly available in https://www.modelarchive.org/, https://doi.org/10.5452/ma-rc0b9 (LeuRS), https://doi.org/10.5452/ma-d28xp (MYO6 pre-powerstroke conformation) and https://doi.org/10.5452/ma-we6d7 (MYO6 Pi release conformation).

## References

[1] Shearer AE, Hildebrand MS, Smith RJH, Adam MP (1999). Hereditary hearing loss and deafness overview. GeneReviews.

[2] Morton CC, Nance WE (2006). Newborn hearing screening—a silent revolution. N. Engl. J. Med..

[3] Wémeau J-L, Kopp P (2017). Pendred syndrome. Best Pract. Res. Clin. Endocrinol. Metab..

[4] Hilgert N, Smith RJH, Van Camp G (2009). Forty-six genes causing nonsyndromic hearing impairment: Which ones should be analyzed in DNA diagnostics?. Mutat. Res..

[5] Feldmann D (2004). Large deletion of the GJB6 gene in deaf patients heterozygous for the GJB2 gene mutation: Genotypic and phenotypic analysis. Am. J. Med. Genet. A.

[6] Snoeckx RL (2005). GJB2 mutations and degree of hearing loss: A multicenter study. Am. J. Hum. Genet..

[7] Del Castillo I (2003). Prevalence and evolutionary origins of the del(GJB6-D13S1830) mutation in the DFNB1 locus in hearing-impaired subjects: A multicenter study. Am. J. Hum. Genet..

[8] Dalamón V (2010). GJB2 and GJB6 genes: Molecular study and identification of novel GJB2 mutations in the hearing-impaired Argentinean population. Audiol. Neurootol..

[9] Diaz-Horta O (2012). Whole-exome sequencing efficiently detects rare mutations in autosomal recessive nonsyndromic hearing loss. PLoS ONE.

[10] Duman D, Tekin M (2012). Autosomal recessive nonsyndromic deafness genes: A review. Front. Biosci..

[11] Atik T (2015). Comprehensive analysis of deafness genes in families with autosomal recessive nonsyndromic hearing loss. PLoS ONE.

[12] Choi HJ (2017). Whole-exome sequencing identified a missense mutation in WFS1 causing low-frequency hearing loss: A case report. BMC Med. Genet..

[13] Shearer AE, Eliot Shearer A, Hildebrand MS, Sloan CM, Smith RJH (2011). Deafness in the genomics era. Hear. Res..

[14] Parzefall T (2017). Whole-exome sequencing to identify the cause of congenital sensorineural hearing loss in carriers of a heterozygous GJB2 mutation. Eur. Arch. Otorhinolaryngol..

[15] Jalilian N (2017). A novel pathogenic variant in the ***MITF*** gene segregating with a unique spectrum of ocular findings in an extended Iranian Waardenburg syndrome kindred. Mol. Syndromol..

[16] Pierce SB (2013). Mutations in LARS2, encoding mitochondrial leucyl-tRNA synthetase, lead to premature ovarian failure and hearing loss in Perrault syndrome. Am. J. Hum. Genet..

[17] Soldà G (2016). First independent replication of the involvement of LARS2 in Perrault syndrome by whole-exome sequencing of an Italian family. J. Hum. Genet..

[18] Avraham KB (1997). Characterization of unconventional MYO6, the human homologue of the gene responsible for deafness in Snell’s waltzer mice. Hum. Mol. Genet..

[19] Buonfiglio P (2020). GJB2 and GJB6 genetic variant curation in an argentinean non-syndromic hearing-impaired cohort. Genes.

[20] Dalamón V (2013). Identification of four novel connexin 26 mutations in non-syndromic deaf patients: Genotype-phenotype analysis in moderate cases. Mol. Biol. Rep..

[21] Miyagawa M, Nishio S-Y, Usami S-I (2012). Prevalence and clinical features of hearing loss patients with CDH23 mutations: A large cohort study. PLoS ONE.

[22] Astuto LM (2002). CDH23 mutation and phenotype heterogeneity: A profile of 107 diverse families with Usher syndrome and nonsyndromic deafness. Am. J. Hum. Genet..

[23] Meng X, Liu X, Li Y, Guo T, Yang L (2020). Correlation between genotype and phenotype in 69 Chinese patients with USH2A mutations: A comparative study of the patients with Usher Syndrome and Nonsyndromic Retinitis Pigmentosa. Acta Ophthalmol..

[24] Yasumoto K, Yokoyama K, Shibata K, Tomita Y, Shibahara S (1995). Microphthalmia-associated transcription factor as a regulator for melanocyte-specific transcription of the human tyrosinase gene. Mol. Cell. Biol..

[25] Zhang Z (2018). A novel variant in MITF in a child from Yunnan-Guizhou Plateau with autosomal dominant inheritance of nonsyndromic hearing loss: A case report. Mol. Med. Rep..

[26] Thongpradit S (2020). MITF variants cause nonsyndromic sensorineural hearing loss with autosomal recessive inheritance. Sci. Rep..

[27] Sun L (2016). Molecular etiology and genotype-phenotype correlation of Chinese Han deaf patients with type I and type II Waardenburg Syndrome. Sci. Rep..

[28] Pandya A (1996). Phenotypic variation in Waardenburg syndrome: mutational heterogeneity, modifier genes or polygenic background?. Hum. Mol. Genet..

[29] DuPai CD, Davies BW, Wilke CO (2020). A systematic analysis of the beta hairpin motif in the protein data bank. bioRxiv.

[30] DuPai CD, Cunningham AL, Conrado AR, Wilke CO, Davies BW (2020). TsrA regulates virulence and intestinal colonization in. mSphere.

[31] Zafar S (2020). Novel mutations in CLPP, LARS2, CDH23, and COL4A5 identified in familial cases of prelingual hearing loss. Genes.

[32] Yan W (2015). Modulation of aminoacylation and editing properties of leucyl-tRNA synthetase by a conserved structural module. J. Biol. Chem..

[33] Miyagawa M, Nishio S-Y, Kumakawa K, Usami S-I (2015). Massively parallel DNA sequencing successfully identified seven families with deafness-associated MYO6 mutations: The mutational spectrum and clinical characteristics. Ann. Otol. Rhinol. Laryngol..

[34] Tian T (2018). Identification of a novel MYO6 mutation associated with autosomal dominant non-syndromic hearing loss in a Chinese family by whole-exome sequencing. Genes Genet. Syst..

[35] Cheng J (2014). Exome sequencing identifies a novel frameshift mutation ofMYO6as the cause of autosomal dominant nonsyndromic hearing loss in a Chinese family. Ann. Hum. Genet..

[36] Hertzano R (2008). A Myo6 mutation destroys coordination between the myosin heads, revealing new functions of myosin VI in the stereocilia of mammalian inner ear hair cells. PLoS Genet..

[37] Oka S-I (2020). Clinical characteristics and in vitro analysis of MYO6 variants causing late-onset progressive hearing loss. Genes.

[38] Mazzoli M (2003). Recommendations for the description of genetic and audiological data for families with nonsyndromic hereditary hearing impairment. Audiol. Med..

[39] Schwarz JM, Cooper DN, Schuelke M, Seelow D (2014). MutationTaster2: mutation prediction for the deep-sequencing age. Nat. Methods.

[40] Adzhubei I, Jordan DM, Sunyaev SR (2013). Predicting functional effect of human missense mutations using PolyPhen-2. Curr. Protoc. Hum. Genet..

[41] Salgado D (2016). UMD-predictor: A high-throughput sequencing compliant system for pathogenicity prediction of any human cDNA substitution. Hum. Mutat..

[42] Ioannidis NM (2016). REVEL: An ensemble method for predicting the pathogenicity of rare missense variants. Am. J. Hum. Genet..

[43] Oza AM (2018). Expert specification of the ACMG/AMP variant interpretation guidelines for genetic hearing loss. Hum. Mutat..

[44] den Dunnen JT (2016). HGVS recommendations for the description of sequence variants: 2016 update. Hum. Mutat..

[45] DiStefano MT (2019). Correction: ClinGen expert clinical validity curation of 164 hearing loss gene–disease pairs. Genet. Med..

[46] Kopanos C (2018). VarSome: The human genomic variant search engine. Bioinformatics.

[47] Kolomenski JE (2020). An update on genetic variants of the NKX2-5. Hum. Mutat..

[48] Di Tommaso P (2011). T-Coffee: A web server for the multiple sequence alignment of protein and RNA sequences using structural information and homology extension. Nucleic Acids Res..

[49] Armougom F (2006). Expresso: automatic incorporation of structural information in multiple sequence alignments using 3D-Coffee. Nucleic Acids Res..

[50] Tamura K (2011). MEGA5: Molecular evolutionary genetics analysis using maximum likelihood, evolutionary distance, and maximum parsimony methods. Mol. Biol. Evol..

[51] Webb B, Sali A (2016). Comparative protein structure modeling using MODELLER. Curr. Protoc. Bioinform..

[52] Sali A, Blundell TL (1993). Comparative protein modelling by satisfaction of spatial restraints. J. Mol. Biol..

[53] Shen M-Y, Sali A (2006). Statistical potential for assessment and prediction of protein structures. Protein Sci..

[54] Studer G (2020). QMEANDisCo—distance constraints applied on model quality estimation. Bioinformatics.

[55] Benkert P, Biasini M, Schwede T (2011). Toward the estimation of the absolute quality of individual protein structure models. Bioinformatics.

[56] Wiederstein M, Sippl MJ (2007). ProSA-web: Interactive web service for the recognition of errors in three-dimensional structures of proteins. Nucleic Acids Res..

[57] Pettersen EF (2004). UCSF Chimera—a visualization system for exploratory research and analysis. J. Comput. Chem..

[58] Shapovalov MV, Dunbrack RL (2011). A smoothed backbone-dependent rotamer library for proteins derived from adaptive kernel density estimates and regressions. Structure.

[59] Bank, R. P. D. RCSB PDB: Homepage. https://www.rcsb.org/.

[60] Fukasawa Y (2015). MitoFates: improved prediction of mitochondrial targeting sequences and their cleavage sites. Mol. Cell. Proteomics.

[61] Unconventional myosin-VI. https://www.uniprot.org/uniprot/Q9UM54#Q9UM54-3.

[62] Wulf SF (2016). Force-producing ADP state of myosin bound to actin. Proc. Natl. Acad. Sci. U. S. A..

[63] Schymkowitz J (2005). The FoldX web server: An online force field. Nucleic Acids Res..

[64] Bruque CD (2016). Structure-based activity prediction of CYP21A2 stability variants: A survey of available gene variations. Sci. Rep..

[65] Maubaret C, Griffoin J-M, Arnaud B, Hamel C (2005). Novel mutations in MYO7A and USH2A in Usher syndrome. Ophthalmic Genet..

[66] Sloan-Heggen CM (2016). Comprehensive genetic testing in the clinical evaluation of 1119 patients with hearing loss. Hum. Genet..

[67] Wattenhofer M (2002). Mutations in the TMPRSS3 gene are a rare cause of childhood nonsyndromic deafness in Caucasian patients. J. Mol. Med..

[68] Eiberg H (2006). Autosomal dominant optic atrophy associated with hearing impairment and impaired glucose regulation caused by a missense mutation in the WFS1 gene. J. Med. Genet..

[69] Bernal S (2003). Mutations in USH2A in Spanish patients with autosomal recessive retinitis pigmentosa: high prevalence and phenotypic variation. J. Med. Genet..

[70] Aller E (2006). Identification of 14 novel mutations in the long isoform of USH2A in Spanish patients with Usher syndrome type II. J. Med. Genet..

[71] Oshima A (2008). Mutation profile of the CDH23 gene in 56 probands with Usher syndrome type I. Hum. Mutat..

[72] Domingo-Gallego A (2021). Clinical utility of genetic testing in early-onset kidney disease: seven genes are the main players. Nephrol. Dial. Transplant.

[73] Plant KE, Green PM, Vetrie D, Flinter FA (1999). Detection of mutations in COL4A5 in patients with Alport syndrome. Hum. Mutat..

[74] Smith CJA, Crock PA, King BR, Meldrum CJ, Scott RJ (2004). Phenotype-genotype correlations in a series of wolfram syndrome families. Diabetes Care.

[75] Plantinga RF (2006). A novel TECTA mutation in a Dutch DFNA8/12 family confirms genotype-phenotype correlation. J. Assoc. Res. Otolaryngol..

[76] Eppsteiner RW (2012). Prediction of cochlear implant performance by genetic mutation: The spiral ganglion hypothesis. Hear. Res..

[77] Zhu M (2003). Mutations in the gamma-actin gene (ACTG1) are associated with dominant progressive deafness (DFNA20/26). Am. J. Hum. Genet..

