# Supplementary material for: Predicting pathogenicity for novel hearing loss mutations based on genetic and protein structure approaches
Source: Sci Rep. 2022 Jan 7;12:301. doi: 10.1038/s41598-021-04081-2 (PMC8741999; doi:10.1038/s41598-021-04081-2)
Supplement: Supplementary file 2 — Supplementary Information 2. [file 41598_2021_4081_MOESM2_ESM.docx]

# “Predicting pathogenicity for novel hearing loss mutations based on genetic and protein structure approaches.” Paula I. Buonfiglio†, Carlos D. Bruque†, Vanesa Lotersztein, Leonela Luce, Florencia Giliberto, Sebastián Menazzi, Liliana Francipane, Bibiana Paoli, Ernesto Goldschmidt, Ana Belén Elgoyhen^1^, Viviana Dalamón*

**Supplementary Table S1: 183 deafness susceptibility genes analysed in patients**

| **Recessive inheritance genes:** | ADCY1, ATP2B2, BDP1, BSND, CABP2, CDC14A, CDH23, CEACAM16, CIB2, CLDN9, CLDN14, CLIC5, CLPP, COL11A2, COMT2, DCDC2, ELMOD3, EPS8, EPS8L2, ESPN, ESRP1 ESRRB, FAM65B, GAB1, GIPC3, GJB2, GJB3, GJB6, GPSM2, GRAP, GRXCR1, GRXCR2, HGF, ILDR1, KARS, LHFPL5, LOXHD1, LRTOMT, MARVELD2, MET, MPZL2, MSRB3, MYO3A, MYO6, MYO7A, MYO15A, NARS2, OTOA, OTOF, OTOG, OTOGL, PPIP5K2, PCDH15, PDZD7, PJVK, PNPT1, PTPRQ, RDX, ROR1, S1PR2, SERPINB6, SLC22A4, SLC26A4, SLC26A5, SPNS2, STRC, SYNE4, TBC1D24, TECTA, TMC1, TMEM132E, TMIE, TMPRSS3, TPRN, TRIOBP, TSPEAR, USH1C, WHRN, WBP2. |
| --- | --- |
| **Dominant inheritance genes:** | ACTG1, ATP2B2, CCDC50, CD164, COCH, COL11A1, COL11A2, CRYM, DIABLO, DIAPH1, DIAPH3, DMXL2, DSPP, EYA4, GJB2, GJB3, GJB6, GRHL2, GSMDE, HOMER2, IFNLR1, KCNQ4, LMX1A, MCM2, MYH14, MYH9, MYO3A, MYO6, MYO7A, NLRP3, OSBPL2, PDE1C, PLS1, P2RX2, POU4F3, REST, SCD5, SIX1, SLC12A2, SLC17A8, TBC1D24, TECTA, TECTB, TMC1, TNC, TJP2, TRRAP, WFS1. |
| **X-linked genes:** | COL4A6, POU3F4, PRPS1, SMPX, NDP. |
| **Micro-RNAs:** | miR-96 |
| **Syndromic Deafness**: | SLC26A4, KCNJ10, FOXI16, CDH23, CIB2, CLRN1, HARS, GPR98, MYO7A, PCDH15, PDZD7, USH1C, USH1G, USH2A, WHRN, ALMS1, EYA1, SIX1, SIX5, AIFM1, KCEN1, KCNQ1, WFS1, CISD2, COL4A3, COL4A4, COL4A5, TCOF1, POLR1D, POLR1C, PAX3, MITF, SNAI2, KITLG, SOX10, EDNRB, EDN3, CLPP, HARS2, HSD17B4, LARS2, C10orf2 (TWNK), COL2A1, COL11A1, COL11A2, COL9A1, LOXL3, COL9A2, SLITRK6, OPA1, CEP78, CEP250, CACNA1D, GPSM2 GATA3, ATP6V1B1, GATA3, TBX1, TIMM8A, FGF3, PEX1, PEX6, FGFR1, FGFR2, [PCARE](https://www.genenames.org/tools/search/#!/genes?query=PCARE), CATSPER2, CHD7, SEMA3E, NDP. |
