# Supplementary material for: Predicting pathogenicity for novel hearing loss mutations based on genetic and protein structure approaches
Source: Sci Rep. 2022 Jan 7;12:301. doi: 10.1038/s41598-021-04081-2 (PMC8741999; doi:10.1038/s41598-021-04081-2)
Supplement: Supplementary file 4 — Supplementary Information 4. [file 41598_2021_4081_MOESM4_ESM.docx]

**Supplementary Table S 3: X-Ray crystallography structure evaluated from PDB database to MYO6 protein.**

|  | 4PFP | 4PFO | 4PJN | 2V26 | 4DBR | 4E7Z |
| --- | --- | --- | --- | --- | --- | --- |
| Method | X-ray  crystallography | X-ray  crystallography | X-ray  crystallography | X-ray  crystallography | X-ray  crystallography | X-ray  crystallography |
| Resolution [Å] | 2.32 Å | 1.75 | 2 | 1.75 | 1.95 | 2.30 |
| R-Value  (Free // Work) | 0.229 / 0.189 | 0.192/0.169 | 0.207/0.170 | 0.227/0.202 | 0.216/0.174 | 0.231/0.176 |
| Chains | A,C | A | A | A | A | A,B |
| Length | 788 | 788 | 788 | 784 | 786 | 798 |
| % Identity / Similarity  with all structure  CW | 97.78% / 98.43% | 98.14% / 98.8 % | 98.02% / 98.68% | 98.25% / 98.92% | 97.6% / 98.27% | 98.15% / 98.81% |
| % identity / Similarity with the alignment to generated model | 98.04% / 98.69% | 98.14% / 98.8% | 98.02% / 98.68% | 98.12% / 98.79% | 97.74% / 98.4% | 98.15% / 98.81% |
| pH | 6.5 | 8.5 | 7.5 | 6.75 | 8 | 6.75 |
| Percent Solvent Content | 51.76 | 50.37 | 51.12 | 42.8 | 49.94 | 52 |
| Protein | Myosin VI motor domain in the Pi release state (with Pi) space group P21 | Myosin VI motor domain in the Pi release state, space group P21212 | Myosin VI motor domain in the Pi release state, space group P212121 - shortly soaked with PO4 | Myosin VI (MD) pre-powerstroke state (Mg.ADP.VO4) | Myosin VI D179Y (MD) pre-powerstroke state | Myosin VI (MD) pre-powerstroke state, P21 crystal form |
| Unique Ligand |  | ADP, GOL, MG | ADP, GOL, MG, PO4 | ADP, EDO, MG, SO4, VO4 | ADP, EDO, MG, VO4 | ADP, GOL, MG, VO4 |
| Mutation | 0 | 0 | 0 | 0 | 1 (D176Y) | 0 |
| Conformation | PI release | PI release | PI release | pre-powerstroke | pre-powerstroke | pre-powerstroke |
| Organism | Sus scrofa | Sus scrofa | Sus scrofa | Sus scrofa | Sus scrofa | Sus scrofa |
| PMID | 25936506 | 25936506 | 25936506 | 17956731 | To be published | 22940248 |
